# Performance and Recovery of Well-Trained Younger and Older Athletes during Different HIIT Protocols

**DOI:** 10.3390/sports10010009

**Published:** 2022-01-05

**Authors:** Laura Hottenrott, Martin Möhle, Sarah Feichtinger, Sascha Ketelhut, Oliver Stoll, Kuno Hottenrott

**Affiliations:** 1 Institute of Performance Diagnostics and Health Promotion (ILUG), Martin-Luther-University Halle-Wittenberg, 06108 Halle, Germany; sarah.feichtinger@ilug.de (S.F.); oliver.stoll@sport.uni-halle.de (O.S.); kuno.hottenrott@sport.uni-halle.de (K.H.); 2 Institute of Sport Science, Martin-Luther-University Halle-Wittenberg, 06108 Halle, Germany; martin.moehle@sport.uni-halle.de (M.M.); sascha.ketelhut@sport.uni-halle.de (S.K.)

**Keywords:** aging, master athletes, endurance exercise Wingate test, HIIT, interval training, cycling, recovery, lactate, heart rate recovery

## Abstract

Due to physiological and morphological differences, younger and older athletes may recover differently from training loads. High-intensity interval training (HIIT) protocols are useful for studying the progression of recovery. It was the objective of this study to determine age differences in performance and recovery following different HIIT protocols. Methods: 12 younger (24.5 ± 3.7 years) and 12 older (47.3 ± 8.6 years) well-trained cyclists and triathletes took part in this study. Between the age groups there were no significant differences in relative peak power to fat-free mass, maximal heart rate (HR), training volume, and VO_2max_-percentiles (%). Participants performed different HIIT protocols consisting of 4 × 30 s Wingate tests with different active rest intervals (1, 3, or 10 min). Peak and average power, lactate, HR, respiratory exchange ratio (RER), subjective rating of perceived exertion (RPE), and recovery (Total Quality Recovery scale, TQR) were assessed. Results: During the different HIIT protocols, metabolic, cardiovascular, and subjective recovery were similar between the two groups. No significant differences were found in average lactate concentration, peak and average power, fatigue (%), %HR_max_, RER, RPE, and TQR values between the groups (*p* > 0.05). Conclusion: The findings of this study indicate that recovery following HIIT does not differ between the two age groups. Furthermore, older and younger participants displayed similar lactate kinetics after the intermittent exercise protocols.

## 1. Introduction

Recovery from exercise is essential for continuous performance improvement [1,2]. Due to morphological and physiological changes occurring during aging and age-related alterations in performance capacity, younger and older athletes may recover at different rates from physical exercise and training loads [3,4,5].

Age-related physiological and morphological changes in the muscular system include the selective loss of fast muscle fibers and motor units, along with a decrease in muscle cross-sectional area and number of satellite cells, and a change in muscle architecture [6,7]. Maximum oxygen uptake decreases in both untrained and trained older subjects [7]. However, trained subjects with a higher physical fitness level are able to maintain this higher level compared with untrained subjects [8].

It is well established that maximal heart rate (HR_max_) decreases with age and is independent of sex [9]. The maximum lactic acid production rate, which is determined by the performance capacity or trainability of the fast muscle fibers [10], decreases with age [11]. Although peak blood lactate levels of trained subjects are considerably higher than those reported for untrained subjects, anaerobic energy production from glycolysis declines with older age. This may be a factor responsible for the deterioration in sprint performance [12].

A decrease in muscle mass and shift towards a more oxidative muscle profile mediated by the atrophy of fast-twitch fibers during aging [6] indicates a reduced creatine phosphate metabolic capacity, in addition to a decreased rate lactic acid formation and glycolysis. It may also be possible that the anaerobic energy output decreases with aging due to a reduction in important glycolytic enzymes, particularly phosphofructokinase [13]. Furthermore, glucose transporter (GLUT-4) levels decrease with aging, reducing glucose transport efficacy, possibly affecting performance and recovery in older athletes [14]. Therefore, training adaptation, decline in performance, and recovery in the process of aging varies among different sports and intensities of exercise. According to Fell and Williams [3], an older athlete may require a more extended post-exercise recovery period compared to a younger athlete with a similar performance level when applying the same training load. The extent to which age-related physiological and morphological differences affect recovery in aging athletes during high-intensity interval training (HIIT) sessions with different recovery periods has not yet been investigated.

HIIT sessions are used to improve maximum oxygen uptake (VO_2max_) and endurance performance in high-performance and recreational sports [15,16,17]. High-intensity interval training (HIIT) protocols are variable in their design and can be different regarding the number of repetitions, duration of intervals, intensity, and recovery time between interval bouts, thereby pursuing different training goals [18,19]. Postexercise recovery is a multifaceted (e.g., psychological, physiological) restorative process and an essential component of exercise training. Recovery is crucial to allow for continuous performance development [1,2,20]. The length of recovery time does not only influence the maximal performance during each exercise bout, but also the overall organismic stress [21,22,23]. Due to their intermittent character, HIIT sessions are useful for studying the progression of recovery.

Recovery following repeated sprint and endurance exercise is different in children and adults. Children have a shorter lactate half-life as a result of their lower maximal lactate concentrations, and a faster heart rate recovery (HRR) and respiratory recovery compared to adults [21,24,25,26,27].

Most studies on post-exercise recovery have either compared children and adults or younger and older athletes with different performance levels [5]. Studies with a different level of performance or training status revealed delayed recovery of VO_2_ and VCO_2_ for master athletes compared to adults [28].

Studies on recovery following continuous endurance exercise (running competition) in younger and older athletes (masters) with matching performance levels (VO_2max_) revealed a delayed muscular recovery and greater muscular damage during recovery in masters (45.9 ± 5.9 years) compared to younger athletes (30.5 ± 7 years) [4]. However, no studies have been conducted on recovery during and after high-intensity intermittent exercise in younger and older adults with matched performance levels.

The Wingate anaerobic test (WAnT) comprising four 30 s maximal efforts on a cycle ergometer allows for continuous measurement and recording of heart rate (HR), oxygen uptake, and power [29,30,31,32]. The length of 30 s for short intervals is suitable for the evaluation of anaerobic performance, as shown in many studies on maximal exercise [21,23,31,32,33]. Furthermore, this allows for a differentiated and comprehensive discussion of the findings of this present investigation with previous findings, and for a derivation of practical applications for athletes and coaches.

HIIT is used in elite sports, fitness sports, and recreational sports in a number of ways, and the training design of HIIT sessions, especially the duration of recovery phases, has a direct impact on performance development. However, to date, no comprehensive evaluation has been conducted on whether younger and older athletes respond and recover in a similar manner. Therefore, this study examined a younger and an older group of athletes whose physical performance was comparable. This has not been taken into account in previous studies, in which older athletes typically had a lower performance level and, as a result, their recovery was reduced. However, this study compared the recovery of younger and older athletes with matched physical performance levels.

The aim of this study was to examine possible age-specific differences in cardiovascular, metabolic, and subjective recovery during HIIT with active recovery times of different durations between intervals. Moreover, the study investigated whether different recovery times influence the performance of younger and older athletes during HIIT.

## 2. Materials and Methods

### 2.1. Participants

Two groups consisting of 12 younger (mean age: 24.5 ± 3.7 years; 8 men, 4 women) and 12 older athletes (mean age: 47.3 ± 8.6 years; 8 men, 4 women) took part in this study. For at least six months prior to taking part in the study, all athletes were required to cycle for at least 6 h/week and have a VO_2max_ above the 80th percentile [34]. The baseline values of the 24 athletes are shown in Table 1. There were no significant differences in height, body mass, and fat-free mass (FFM), or in the performance-related parameters HR_max_, relative peak power output to FFM, and weekly training volume between younger and older athletes.

Younger and older athletes had a comparable maximal aerobic performance capacity according to sex and age-specific VO_2max_ percentiles [34]. The bioimpedance values showed age differences for body fat but not for FFM. There were no significant differences in the bioimpedance data of the athletes between the testing days.

**Table 1:** Anthropometric data, exercise performance parameters, and maximal heart rate (HR_max_), of the athletes at baseline measurements. Data are means ± SD.

Parameter	Younger Athletes (*n* = 12)	Older Athletes (*n* = 12)	*p*-Values
Age (years)	24.5 ± 3.7	47.3 ± 8.6	<0.001
Height (m)	1.76 ± 0.11	1.72 ± 0.11	0.423
Body mass (kg)	65.9 ± 10.9	70.8 ± 11.0	0.281
BMI (kg/m^2^)	21.1 ± 1.8	23.7 ± 2.2	0.004
Body fat (%)	9.8 ± 6.3	14.9 ± 6.1	0.011
FFM (kg)	59.8 ± 12.0	59.3 ± 9.5	0.901
VO_2max_ (mL/min/kg)	56.7 ± 7.0	49.2 ± 6.4	0.011
HR_max_ (min^−1^)	179.2 ± 11.1	174.9 ± 11.7	0.371
Peak Power (W/kg)	5.24 ± 0.58	4.66 ± 0.43	0.011
Peak Power (W/kg_FFM_)	5.79 ± 0.47	5.60 ± 0.71	0.122
VO_2max_-percentile (%)	95.4 ± 5.2	93.1 ± 5.8	0.326
Training (h/week)	8.73 ± 3.62	8.37 ± 2.28	0.627

This investigation was approved by the Martin-Luther-University Halle-Wittenberg Ethics Committee (Reference code: 2019-094) and conducted in accordance with the Declaration of Helsinki.

### 2.2. Test Protocol

Each test took place under standardized conditions of a 20 °C lab temperature and 55% relative humidity. Subjects reported to the laboratory on four occasions. They had to be in a recovered and hydrated state after having fasted for at least two hours. Additionally, they had to abstain from strenuous exercise for 48 h prior to all tests. Every athlete was tested again at the same time of day, and all cycling tests were conducted on the same cycling ergometer during each visit. During the entire course of the study, all athletes agreed to maintain their usual dietary habits and to document their daily training load.

On the first of four visits, baseline assessments took place. Subjects completed a medical questionnaire and indicated they had not taken any supplements or medication that could influence the results. Body composition (body mass, FFM and body fat) was determined after 20 min in the supine resting position using a Bio Impedance Analyzer (Data Input GmbH, Pöcking, Germany). Then, a Metalyzer 3B (Cortex, Leipzig, Germany) was applied in an incremental step test until voluntary exhaustion on an elite bicycle ergometer (E 2000s, FES, Berlin, Germany) to determine the athlete’s aerobic fitness in terms of oxygen uptake. This test started with a warm-up over eight minutes at 70 W for female athletes and at 100 W for male athletes on the cycling ergometer. After the warm-up, athletes completed the VO_2max_ test [35,36]. Thereby, all athletes started with a resistance of 70 W and, each minute, the power increased by 30 W. For all athletes, the cadence was set at 80–90 rpm throughout the entire test.

**Figure 1:**
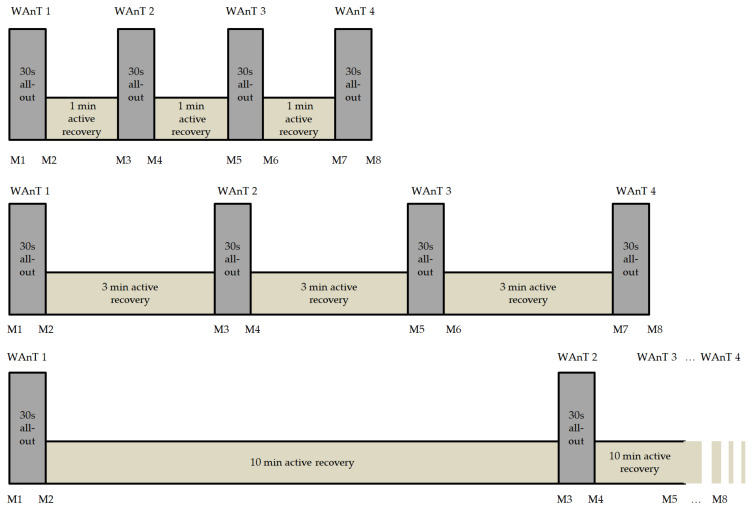
Testing protocol of the Wingate anaerobic tests (WAnT) during high-intensity interval training (HIIT). The three different protocols with four Wingate tests each, regarding the three active recovery times at 70 W for females and 100 W for males (1, 3, or 10 min active recovery) were performed in a randomized order with one week recovery between (M1–M8: measurement points).

One week following the baseline test, athletes completed the first of three HIIT sessions. Thereby, a 30 s WAnT was performed four times, separated by different active recovery periods at each visit (1, 3, or 10 min) as displayed in Figure 1. The athletes performed the three different HIIT protocols under standardized conditions in a randomized order regarding the three recovery times (1, 3, or 10 min). Each test was separated by one week of recovery. The power for the warm-up, active recovery periods, and cool-down was set at 70 W for female athletes and at 100 W for male athletes, with a cadence of 80–90 rpm. Using 10 µL of blood taken from the ear lobe, lactate levels were measured with the enzymatic-amperometric method (Mueller, model Super GL ambulance, Freital, Germany). Throughout all tests, beat-to-beat (RR) intervals and the HR using an HR monitor (RS800 CX and Polar WearLink W.I.N.D., Polar Electro GmbH, Büttelborn, Germany) and gas exchange using a Metalyzer 3B (Cortex, Leipzig, Germany) were continuously recorded. The subjective rating of exertion and state of recovery were determined using the Rating of Perceived Exertion (RPE) scale [37] and the Total Quality Recovery scale (TQR) [38]. The RPE value of the athletes was assessed after each of the four WAnT intervals, and the TQR value after every active recovery period and every three minutes during the 15 min cool-down.

The testing protocol with the measurement points is shown in Figure 1. The testing protocol was used in an extensive investigation on sex and age differences with different subject groups during HIIT using a consistent study design. The results regarding sex differences have already been published [23]. For lactate determination, capillary blood was taken before the start of the HIIT protocol after a standardized warm-up and at the measurement points M2, M4, M6, and M8 (Figure 1). The standardized warm-up consisted of 8 min cycling at 70 W for females and 100 W for males, and the cadence was set at 80–90 rpm. The RPE values [35] were recorded at the measuring points M2, M4, M6, and M8. The TQR values [36] were recorded at M3, M5, and M7. Throughout the 10 min active recovery period, blood for lactate determination was also taken. During the 15 min active cool-down, lactate concentration and the TQR rating were determined at 3, 6, 9, 12, and 15 min. Continuous recording of power, ventilatory parameters (breath by breath), and HR (beat to beat interval) took place throughout the entire test period. Intraindividual fatigue, as the respective performance decline within each of the WAnTs over the 30 s duration, was calculated using the formula: %fatigue = (peak powerWAnT − average powerWAnT)/peak powerWAnT × 100) [39].

### 2.3. Statistical Analysis

Descriptive statistics of the data are presented as mean ± standard deviation (SD). Statistical analysis was conducted with IBM SPSS Statistics (version 25, International Business Machines Corporation, Armonk, NY, USA) and a published spreadsheet [40]. A repeated measures two-way ANOVA with Bonferroni corrections for multiple comparisons was used to detect interaction effects if warranted. Univariate post hoc analyses, including one-way ANOVA or two-tailed paired t-tests, were performed with Bonferroni’s correction where appropriate. The level of significance was set at *p* < 0.05.

## 3. Results

### 3.1. Power

The two age groups showed significant differences in the peak power output relative to body mass at baseline (younger: 5.24 ± 0.58 W/kg, older: 4.66 ± 0.43 W/kg (*p* = 0.01) (Table 1). Considering peak power output relative to FFM, there were no significant differences between the age groups (younger: 5.79 ± 0.47 W/kgFFM, older: 5.60 ± 0.71 W/kgFFM (*p* = 0.44)). Table 2 shows the mean and standard deviation of peak power output (PP), average power output (AP), and percentage of fatigue during the different WAnT protocols for the younger and older groups. In PP, significant differences were found between WAnTs one and WAnTs four for both groups in the one-, three-, and ten-minute recovery protocols. AP also declined significantly between WAnTs one and four in both groups throughout the one- and three-minute protocols. In the ten-minute protocol, AP decreased significantly in the younger group only. Fatigue (%) decreased significantly from WAnTs one to four in the HIIT protocol with one minute recovery in the older group and in the ten-minute recovery protocol in the younger groups. The three-minute recovery protocol showed no significant differences in fatigue (%). No significant differences were found between the groups in PP, AP, and fatigue (%) throughout the different HIIT protocols (Table 2).

**Figure 2:**
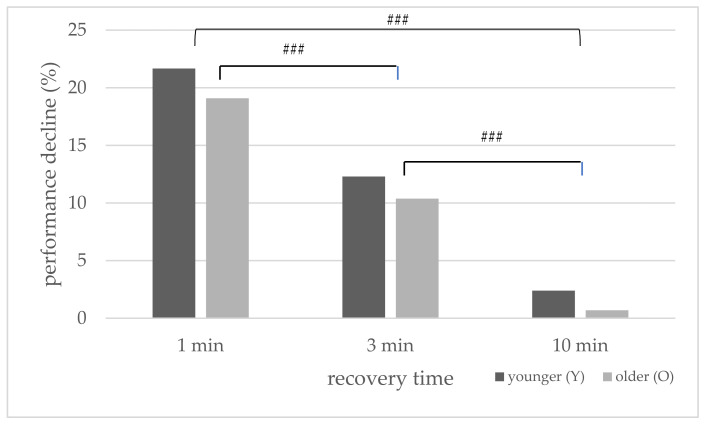
Mean values of performance decline (%) during the three Wingate test (WAnT) protocols with different recovery periods for the younger and older groups ### (*p* < 0.001).

The performance decline (%) for both age groups for the HIIT protocols with one-, three-, and ten-minute recovery is displayed in Figure 2. For both age groups, the overall performance decline was most significant in the one-minute HIIT protocol. The ANOVA neither revealed an interaction effect for performance decline (%) × age (*F*(2,44) = 0.04, *p* = 0.096) nor a main effect for the between-subjects factor (*F*(1,22) = 0.99, *p* = 0.33). There was only a main effect for the within-subject factor (*F*(2,44) = 63.59, *p* < 0.001).

**Figure 3:**
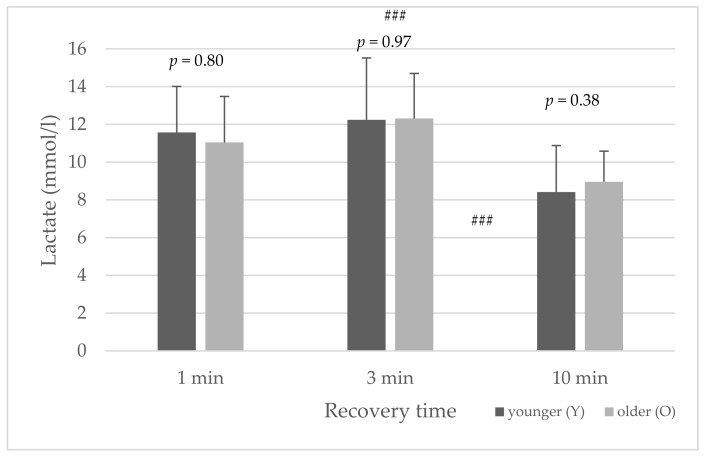
Mean and standard deviation of average lactate concentration (mmol/L) for the younger and older groups ### (*p* < 0.001) between the different protocols (3 to 10, and 1 to 10 min).

**Figure 4:**
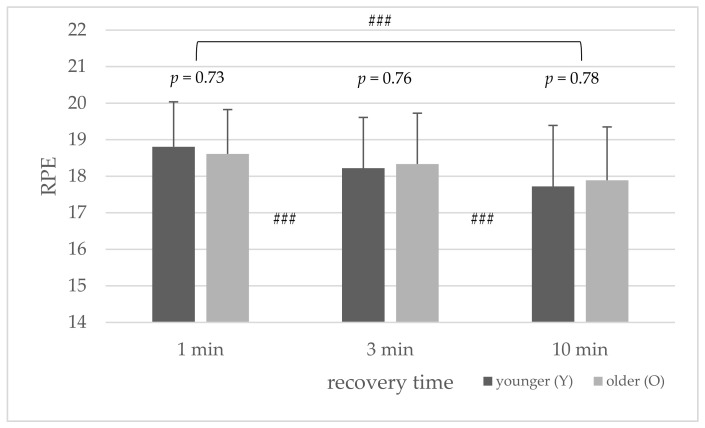
Mean and standard deviation of average rating of perceived exertion (RPE) for the younger and older groups ### (*p* < 0.001) between the different protocols (1 to 3, 3 to 10, and 1 to 10 min).

**Figure 5:**
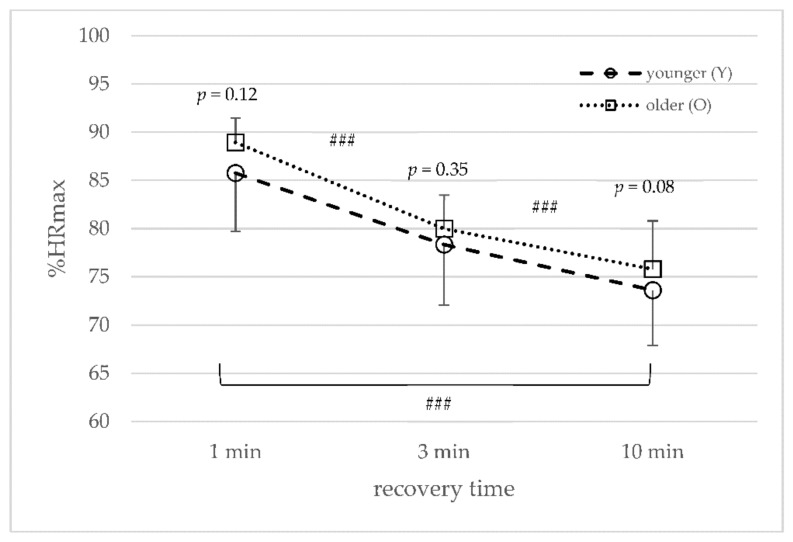
Mean and standard deviation of average percentage of heart rate recovery for the younger and older groups ### (*p* < 0.001) between the different protocols (1 to 3, 3 to 10, and 1 to 10 min).

**Figure 6:**
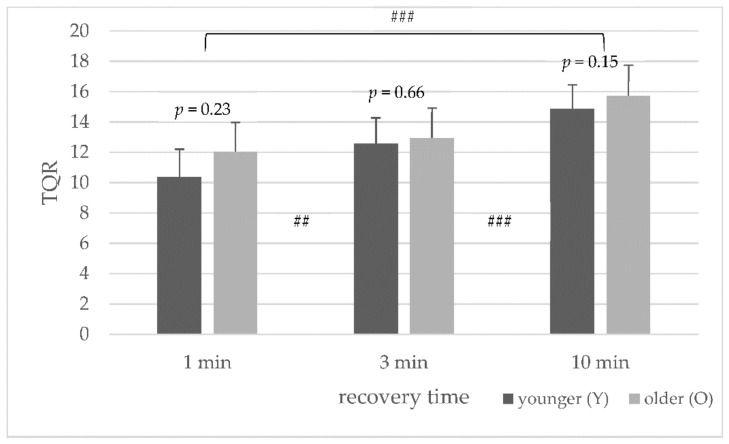
Mean and standard deviation of average perceived recovery (TQR scale) for the younger and older groups ## (*p* < 0.01) and ### (*p* < 0.001) between the different protocols (1 to 3, 3 to 10, and 1 to 10 min).

Figure 3, Figure 4, Figure 5 and Figure 6 show the data following the second, third, and fourth WAnTs in the three different HIIT protocols.

### 3.2. Lactate

The average lactate concentration in the three HIIT protocols showed no significant age differences (Figure 3). For both age groups, the average lactate concentration was highest at a recovery time of three minutes between the WAnTs. The average lactate concentration significantly differed between the recovery time of one to ten minutes (*p* = 0.005) and three to ten minutes (*p* < 0.001) for both age groups, respectively, but not for one to three minutes (*p* = 0.36).

### 3.3. Heart Rate

The average recovery rate for %HRmax at the end of the second, third, and fourth recovery phases were not significant between the younger and older groups, or between the different HIIT protocols (Figure 4). However, the younger group showed a slightly but not significantly higher HR recovery throughout the different HIIT protocols. For both age groups, the %HR_max_ declined with an increase in recovery time for one to three (*p* < 0.001), three to ten (*p* < 0.001), and one to ten minutes (*p* < 0.001) (Figure 4).

### 3.4. Subjective Rating of Perceived Exertion (RPE Scale)

The average RPE values did not differ significantly between the younger and older group in the HIIT protocols (Figure 5). Overall, the RPE values decreased with the increase in recovery time between the WAnTs. Both age groups showed significantly lowered RPE values from one to three minutes (*p* < 0.001), three to ten minutes (*p* < 0.001) and one to ten minutes (*p* < 0.001), respectively.

### 3.5. Subjective Rating of Perceived Recovery (TQR Scale)

No significant age differences in the average TQR values in the HIIT protocols (1, 3, and 10 min recovery) were found (Figure 6). With an increase in recovery time, there was an increase in TQR values. The average TQR values differed significantly for both age groups between one and three minutes (*p* = 0.006), and one and ten minutes (*p* < 0.001), and three and ten minutes of recovery time (*p* < 0.001).

### 3.6. Ventilatory Parameter during Recovery

No significant differences were found in the average respiratory exchange ratio (RER) between the age groups for all measurement points at the end of the recovery periods during all three HIIT protocols (1 min recovery: younger 1.17 ± 0.11, older 1.17 ± 0.08; 3 min recovery: younger 1.05 ± 0.05, older 1.07 ± 0.05; 10 min recovery: younger 0.88 ± 0.02, older 0.87 ± 0.01). There was a significant decrease in RER from one to three minutes of recovery time (*p* = 0.002), and one to ten (*p* < 0.001) and three to ten (*p* < 0.001) minutes of recovery time in both age groups.

## 4. Discussion

There were no statistically significant differences in performance-related parameters VO_2max_ percentile [34] and power (relative PP (W/kg_FFM_)), or HR_max_, attained during the VO_2max_ test between the group of younger and older athletes (Table 1). Thus, both groups displayed comparable relative aerobic performance levels. Furthermore, both groups showed no significant differences in PP, AP, and fatigue (%) throughout the WAnTs in the different HIIT protocols (Table 2).

The main findings of this study were that, during HIIT with repeated 30 s of all-out cycling exercise (WAnT), metabolic, cardiovascular, and subjective recovery was similar between the younger and older athletes. No significant differences were found in the average lactate concentration, peak power, average power, fatigue (%), %HR_max_, RER, RPE, and TQR values between the age groups during the HIIT protocols with 1, 3, and 10 min recovery periods between WAnTs (Table 2 and Figure 3, Figure 4, Figure 5 and Figure 6) (*p* > 0.05).

No significant differences in average lactate values during HIIT between younger and older subjects were detected. This is unexpected considering possible physiological and morphological changes associated with aging, such as a decreased maximum lactic acid production rate [11]. Furthermore, a decrease in muscle mass and shift towards a more oxidative muscle profile mediated by the atrophy of fast-twitch fibers is supposed to occur during aging [6], and indicates a reduced creatine phosphate metabolic capacity and a decreased rate of lactic acid formation and glycolysis. A variation in the response to HIIT using the Wingate test between subjects with a high rate of slow-twitch muscle fibers and subjects with a high rate of fast-twitch muscle fibers was examined by Lievens et al. [41]. Power recovered significantly faster in the “slow-twitch” group than in the “fast-twitch” group.

Because muscle biopsies were not taken in this study, we cannot present results for muscle fiber profiles in relation to recovery and performance during HIIT. However, the presented results, showing no age differences in physiological recovery, are in line with Fell et al. [42,43]. Here, too, no differences were found in physiological recovery for a group of nine master cyclists (mean age: 45 years) compared to nine adult cyclists (mean age: 24 years) with similar VO_2max_. Results from three 30 min time trials with similar absolute power output on three consecutive days showed no statistically significant differences for average power, lactate, countermovement jump height, sprint performance, and MVIC between the two age groups. Only perceptual measures for fatigue and soreness were higher, and perceptive recovery was lower in masters from the first to the third time trial. Adults showed no significant change in perceptual measures from the first to the third time trial. These results by Fell et al. [42,43] may suggest that master athletes perceived that they take a longer time to recover, even though they were able to physically recover at a similar rate compared to younger athletes of the same performance level. In the present study, we found no differences in RPE with our focus being on recovery during HIIT and not on the recovery on three consecutive days. In the absence of any published studies with the same or similar study design, and athletes with a similar performance level but different ages, no direct comparisons can be made.

However, results by Silverman and Mazzeo [44] for plasma lactate, glucose, and hormone levels in 24 trained cyclists and 23 untrained men constituting groups of young (trained 22.6 ± 0.8 years, untrained 22.9 ± 1.0 years), middle-aged (trained 46.5 ± 0.9 years, untrained 43.6 ± 1.1 years), and old (trained 63.9 ± 1.8 years, untrained 67.0 ± 2.2 years) subjects are of particular interest in the context of the present findings. Silverman and Mazzeo [44] had subjects perform a maximal incremental cycling test and a 45 min submaximal exercise test in which peak oxygen consumption was lower with older age. However, the trained groups with higher values for VO_2peak_ compared to the untrained groups had increased hormonal responses (cortisol, norepinephrine, epinephrine, and human growth hormone), as determined by plasma concentrations, to submaximal and maximal exercise in every age group. Furthermore, during 45 min of submaximal exercise, older trained individuals achieved results for all assessed hormone levels that were comparable to those of their younger counterparts, and showed significantly greater results compared to the untrained groups (both young and middle-aged groups). Comparable results were found during the maximal exercise testing in which older trained individuals demonstrated greater hormonal responses than the younger untrained group. Thus, this would suggest that, for a particular metabolic stress as induced in the study of Silverman and Mazzeo [44], neuroendocrine responses are enhanced with higher fitness levels and training throughout life may attenuate the decline in neuroendocrine function, which is in support of the present findings during HIIT.

Regarding %HR_max_, the present results found a similar response in the two age groups, even though the values were lower, and the changes between the 1, 3, and 10 min recovery time HIIT protocols were identical (Figure 4). It is well established that HR_max_ decreases with age [9], which is supported by the present results (Table 1). Accordingly, the HR was lower during the HIIT protocols; however, the course of change during each HIIT protocol and between the different HIIT protocols was similar. These results are supported by Darr et al. [45], who investigated HRR following a maximal incremental cycling test until exhaustion in trained and untrained groups of different ages. Darr et al. [45] divided 20 male cyclists based on their age and VO_2peak_ into four groups of young trained, old trained, young untrained, and old untrained. Although they found no differences in HRR in the trained age groups, they found differences in HRR in the untrained age groups. HRR was delayed in untrained compared to trained subjects. HRR was about 6 beats per minute faster in trained subjects compared to the untrained (VO_2peak_ 60 vs. 40 mL/min/kg), while no age effect of HRR was observed. Taken together with the present findings, the course of the %HR_max_ and HRR during intensive cycling exercise seems to be independent of age but dependent on training status.

When comparing the three different HIIT protocols, the measured parameters differed depending on the length of the recovery time between the 30 s sprints. For both age groups, the overall performance decline was greatest in the HIIT protocol with the shortest recovery time and decreased with the increase in recovery time (Figure 2).

In both age groups, AP also declined between WAnTs one and four for the one- and three-minute protocols (Table 2). However, in the ten-minute protocol, AP decreased significantly in the younger group only. The fatigue (%) decreased significantly from WAnTs one to four in the HIIT protocol with one minute recovery time for the older and ten-minute recovery for the younger group. It is difficult to find a possible explanation and draw conclusions for these differences because no previous studies with a comparable study design have been conducted, and except for the power data, no age differences between the different HIIT protocols have been found. With increasing age, there is a decrease in muscle mass and atrophy of fast-twitch muscle fibers, which, in turn, results in a shift to a more oxidative muscle profile [6,7]. Due to these altered muscle properties, there may be a decreased metabolic capacity of creatine phosphate, which may lead to negative effects on maximal power output after a short recovery time (1 min) during high-intensity exercise. Furthermore, anaerobic energy output may have decreased with ageing, due to a reduction in glycolytic enzymes, particularly phosphofructokinase [6,7,13].

For both age groups, the average lactate concentration was highest in the HIIT protocol with the recovery time of three minutes between the WAnTs. The subjective recovery (Figure 6) increases with the length of the recovery time, and the decrease in power is lower at 3 min than at 1 min (Figure 2), and the power (peak power) is higher at 3 min (Table 2). Thus, more fast muscle fibers could be utilized, and the lactate concentration increased at 3 min, and during the 3 min recovery period, the lactate concentration could not be completely dissipated as during the 10 min recovery period.

## 5. Limitations

Some limitations must be considered when interpreting the results of this study. Although the performance level was similar in both groups, evaluating men and women together in terms of metabolic and cardiovascular effects of HIIT protocols is a limitation. Moreover, females were not tested during a standardized phase during their menstrual cycle. Future studies should further investigate age differences in male- and female-only groups. Additionally, the age gap between the two groups was 22.8 years. Future studies should try to find two age groups with a comparable performance level but an older master athletes group. Furthermore, it is a general limitation that a clear definition for the age of master athletes across different sports is missing. This study investigated HIIT with active recovery phases between exercise bouts. It would be interesting to replicate this study design with passive recovery phases between intervals.

## 6. Conclusions

The present study is the first to show how recovery during and after HIIT of younger and older athletes with similar performance levels compares. This study is particularly unique due to the fact that the data were obtained in well-trained athletes following three HIIT protocols with different recovery times in a randomized order.

The present study revealed novel findings in regards to lactate, heart rate, and subjective recovery during short high-intensity intervals (HIIT) with rest intervals of different durations in young and older well-trained cyclists with comparable aerobic performance levels (VO_2max_ > 80th percentile [34], and relative power). This study indicates that recovery at the metabolic level following HIIT does not differ between the two age groups. Instead, it seems that the trainability of the organism is maintained. With respect to lactate, trainability at older age further ensures that metabolic processes occur in the same manner as in younger subjects. Although a direct comparison with other studies is difficult due to the limited amount of comparable study designs, parallels can be drawn from previous results [5,21,23,42,43,44,45].

The present results are particularly valuable as studies on HIIT are mostly conducted on young athletes, and it is purely speculative to assume that training recommendations apply equally to all age groups. However, based on the current results, HIIT can achieve similar training goals in younger and older athletes, considering that the athletes’ performance levels are comparable. These findings also show that an active recovery of well over 3 to 10 min is recommendable for both younger and older athletes in order to achieve high power output in each interval and to minimize fatigue-induced power loss. According to the group of subjects investigated, these statements are applicable to athletes up to about 50 years of age. Above this range, the extent to which the trainability during HIIT identified in this study is maintained at an even older age, for example, up to the age of 75, should be further examined. The results of the present investigation were obtained using HIIT sessions consisting of four all-out sprints of 30 s. How the current findings relate to long HIIT intervals of 2–5 min [46] remains to be investigated.

## Figures and Tables

**Table 2:** Mean and standard deviation of peak power, average power, and fatigue during Wingate tests (WAnTs) with different recovery times for younger (Y) and older (O) athletes * *p* < 0.05, ** *p* < 0.01, *** *p* < 0.001 between T1–T2, T2–T3, and T3–T4).

Parameter	Recovery	Age	WAnT 1	WAnT 2	WAnT 3	WAnT 4	T1–T4
Peak Power (W)	1 min	Y	734.8 ± 225.4	583.5 ± 157.3 ***	554.4 ± 140.3 *	530.5 ± 131.7	*p* < 0.001
		O	724.1 ± 181.2	579.8 ± 145.4 ***	550.3 ± 135.3	533.7 ± 145.5	*p* < 0.001
Av. Power (W)		Y	535.8 ± 192.3	454.8 ± 157.5 ***	422.4 ± 141.3 ***	410.1 ± 135.3 *	*p* < 0.001
		O	485.8 ± 126.3	418.4 ± 110.2 ***	402.3 ± 109.5 *	395.3 ± 119.0	*p* < 0.001
Fatigue (%)		Y	27.3 ± 12.1	23.3 ± 9.8 *	25.1 ± 9.0	23.6 ± 10.9	*p* = 0.11
		O	32.7 ± 9.0	27.7 ± 9.6 *	26.8 ± 12.0	26.4 ± 8.2	*p* = 0.01
Peak Power (W)	3 min	Y	739.4 ± 218.5	663.8 ± 183.4 **	637.3 ± 188.8 *	608.8 ± 160.7 *	*p* < 0.001
		O	731.0 ± 130.5	671.8 ± 151.0 **	629.1 ± 137.7 *	617.6 ± 129.5	*p* < 0.001
Av. Power (W)		Y	537.7 ± 202.6	498.4 ± 180.7 **	476.8 ± 172.0 ***	465.3 ± 166.2 **	*p* < 0.001
		O	499.8 ± 134.4	471.2 ± 125.7 ***	447.3 ± 117.4 **	445.8 ± 115.3	*p* < 0.001
Fatigue (%)		Y	28.4 ± 11.1	26.0 ± 11.9	26.3 ± 9.7	25.0 ± 11.0	*p* = 0.09
		O	32.4 ± 10.7	29.9 ± 11.7	29.4 ± 8.8	28.1 ± 10.6	*p* = 0.06
Peak Power (W)	10 min	Y	749.8 ± 217.3	692.0 ± 207.0 **	682.1 ± 216.9	678.2 ± 194.4	*p* < 0.001
		O	728.8 ± 187.6	709.6 ± 156.9	691.1 ± 149.7	672.8 ± 150.3	*p* = 0.04
Av. Power (W)		Y	536.2 ± 202.1	526.0 ± 193.2	523.2 ± 193.3	520.1 ± 188.9	*p* = 0.03
		O	500.4 ± 141.4	504.0 ± 131.8	499.3 ± 130.7	493.3 ± 129.5	*p* = 0.43
Fatigue (%)		Y	29.7 ± 10.6	25.1 ± 9.6 **	24.3 ± 8.3	24.7 ± 9.5	*p* < 0.001
		O	31.3 ± 10.1	29.2 ± 9.6	28.1 ± 9.1	26.6 ± 11.8	*p* = 0.07
